# The evolving epidemiology of opioid use disorder: polysubstance use, drug supply transformation, and policy implications

**DOI:** 10.3389/fpubh.2026.1861122

**Published:** 2026-09-09

**Authors:** Naga Koushik Mandapati, Diego Cardenas Tovar, Riya Bindlish, Yesenia Lopez Hernandez, Michele J. Alves, Marianna K. Baum, Saurabh Aggarwal

**Affiliations:** 1 Department of Cellular and Molecular Medicine, Herbert Wertheim College of Medicine, Miami, FL, United States; 2 Department of Biostatistics, Robert Stempel College of Public Health and Social Work, Florida International University, Miami, FL, United States

**Keywords:** epidemiology, fentanyl, harm reduction (HR), nitazenes, opioid use disorder, polysubstance use, synthetic opioids, xylazine

## Abstract

**Background:**

Synthetic Opioids and the rising polysubstance use have caused a substantial change in the epidemiology of opioid use disorder (OUD) in the United States. Current overdose patterns may no longer be adequately explained by traditional opioid-driven theories.

**Methods:**

A structured review was conducted using PRISMA-informed methods. Literature published between 2010 and 2026 was identified through PubMed, Scopus, and Google Scholar using terms related to OUD, fentanyl, polysubstance use, and treatment strategies. Eligible sources included peer-reviewed studies and government reports addressing epidemiology, risk factors, and interventions.

**Results:**

A polysubstance-driven milieu marked by fentanyl monopoly, stimulant co-use, and new adulterants like xylazine and nitazenes has resulted from the opioid crisis’s evolution through several phases. A regional evaluation of South Florida shows how current drug market trends and past prescribing practices converge to increase the risk of overdose. Structural inequities, like unstable finances and limited access to therapy, continue to affect OUD outcomes. Systemic barriers also make evidence-based treatments less effective.

**Conclusion:**

Opioid use disorder needs to be recognized within a wider context of polysubstance risk rather than seen as a solely opioid issue. Successful strategies demand comprehensive policy responses, enhanced harm reduction measures, and flexible surveillance systems that can adapt to rapid shifts in drug availability and usage trends.

## Graphical abstract

**Figure:**
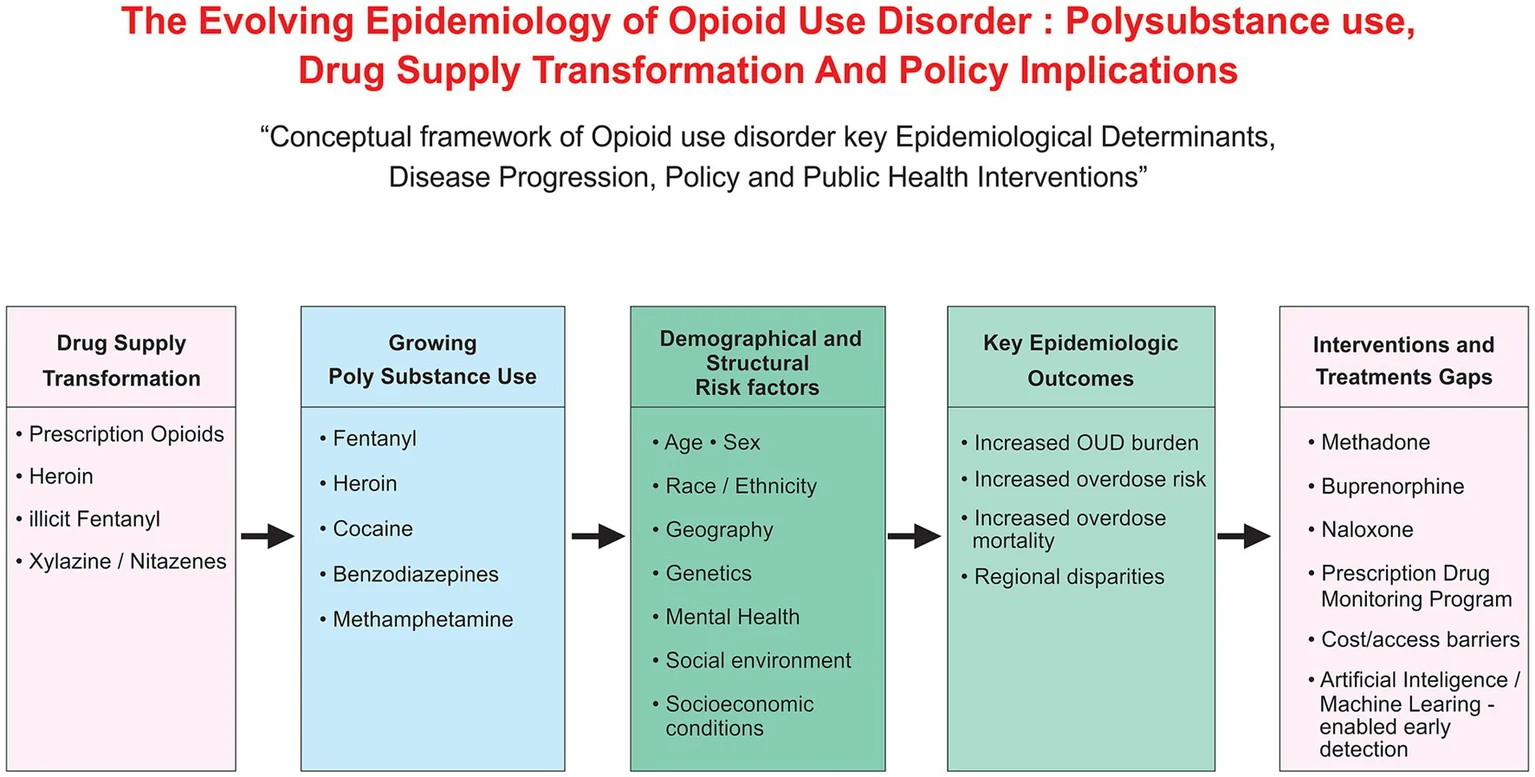

## 1 Introduction

Opioid use disorder (OUD) remains a major and evolving public health crisis in the United States, with substantial morbidity, mortality, and societal costs. Ballreich et al. (1) project the opioid epidemic will cost $412B by 2039, and total $5.8T over 15 years, including $1.8T in healthcare, $3.4T in lost productivity, and $0.7T in broader societal impacts. Incident Medical Opioid Use (IMOU) of new opioid prescriptions for acute and chronic pain is expected to drive 3.3 million new OUD cases, 110,000 overdose deaths, and $890B in costs over the same period. Reducing IMOU by substituting non-opioid therapies could significantly mitigate this burden: a 10% shift may prevent 323,000 OUD cases and 11,000 deaths while saving $88B, while a 25% shift could avert 808,000 cases, 27,000 deaths, and $221B in costs (1). Although the crisis initially emerged in the context of widespread opioid prescription for pain, it has since evolved into a more complex epidemic driven by illicit synthetic opioids, polysubstance use, and persistent barriers to effective treatment. These changes highlight the need for a comprehensive understanding of the factors that contribute to OUD and sustain its impact across diverse populations and regions.

Patterns of opioid use and prescription practices have played a central role in influencing the trajectory of the epidemic (2). Variability in prescribing behaviors, insufficient oversight, and the normalization of opioid-based pain treatment created conditions that facilitated misuse and dependence (3). These prescribing patterns intersect with demographic and individual risk factors, including socioeconomic instability, mental health conditions, exposure to trauma, and biological vulnerability, collectively raising the risk of developing OUD (4). Besides individual health consequences, OUD exerts profound effects on medical care systems and communities. Augmented healthcare utilization, reduced productivity, and strain on public health infrastructure reflect the disorder’s broader societal impact. Certain regions have been disproportionately affected, with South Florida representing a considerable example because of its historical concentration of high-volume pain clinics and its role in the early expansion of opioid distribution networks (5). The consequence of these practices persists in influencing regional patterns of opioid misuse and overdose (6).

Emerging evidence also accentuates the role of genetic susceptibility in addiction vulnerability (7). Interactions between genetic susceptibilities and environmental exposures influence the brain’s reward circuitry, particularly dopaminergic transmission pathways, causing differences in individual responses to opioid exposure (8). Recognizing this biological heterogeneity is essential to developing more targeted and efficient prevention and treatment strategies. As the opioid crisis continues to evolve, prevention and treatment approaches must adapt accordingly. In addition to established pharmacologic and behavioral interventions, advances in technology, such as artificial intelligence and machine learning, provide new opportunities to improve risk identification, support safer prescribing, and boost early intervention.

This review analyzes OUD through an analysis of opioid use patterns and prescribing practices, demographic and biological risk factors, societal and health impacts, South Florida’s past role in the opioid crisis, and emerging strategies for prevention and treatment. By uniting these perspectives, the paper aims to provide an inclusive framework for understanding OUD and to guide future efforts to reduce its ongoing impact.

## 2 Methods

This structured narrative review used a PRISMA-informed selection process. PubMed, Scopus, and Google Scholar were searched for publications from 2010 to 2026 using terms related to opioid use disorder, fentanyl, polysubstance use, xylazine, nitazenes, epidemiology, treatment, and policy. Eligible sources included peer-reviewed studies, systematic reviews, and official government reports addressing OUD epidemiology, risk factors, treatment, prevention, or policy. Non-peer-reviewed sources, non-government reports, animal studies, unrelated substance use disorders, and records lacking sufficient relevance were excluded.

**Figure 1:**
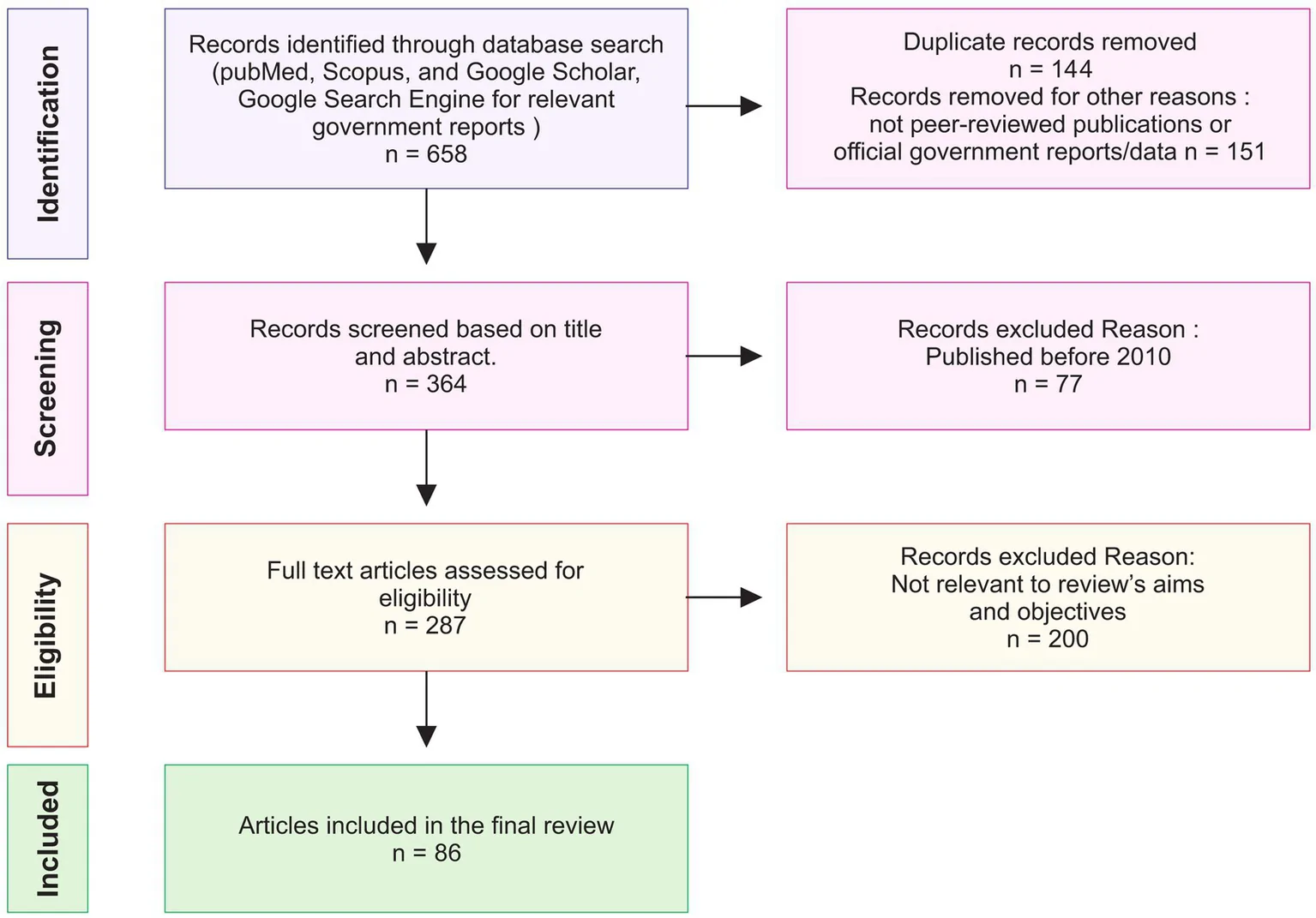
PRISMA flow chart.

The search identified 658 records. After removing 144 duplicates and 151 ineligible records, 364 records were screened by title and abstract. Seventy-seven records published before 2010 were excluded, and 287 full-text articles were assessed. Of these, 200 were excluded for lack of relevance, leaving 86 publications for narrative synthesis (Figure 1). Data were synthesized narratively, with emphasis on emerging trends in polysubstance use, regional patterns, including South Florida, and evolving prevention and treatment strategies.

## 3 Results

### 3.1 Evolution of the opioid crisis

The opioid crisis in the United States has occurred in four distinct waves (Table 1). The first wave, which began in the 1990s and peaked around 2011, was associated with the overprescription of opioid pills and the expansion of extended-release and long-acting opioid formulations, which increased the availability of high-dose opioid products. Some of these formulations could be manipulated by crushing, insufflation, or injection, which increased their potential for misuse. However, the rise in prescription opioid use was not attributable only to prescribing practices. Structural factors, including an aging population, increasing reports of pain and disability, economic distress, declining social cohesion, and psychological distress, may also have increased vulnerability to opioid misuse (2, 9). The second wave, which was defined by increased heroin use, began in the mid-2000s, with the deaths related to heroin overdose accelerating in 2011 (9). The prescription opioid and heroin waves were closely interconnected because some individuals who had developed dependence on prescription opioids transitioned to heroin when pills became more expensive or difficult to obtain. The availability of relatively inexpensive and high-purity heroin may have facilitated this transition. The reformulation of OxyContin into an abuse-deterrent formulation in late 2010 may also have contributed to heroin initiation among a limited proportion of individuals already at risk, although this relationship should not be interpreted as the sole cause of the increase in heroin use (9). The third wave began approximately 2014 and was characterized by a dramatic rise in overdose deaths involving illicitly manufactured fentanyl and fentanyl analogs (9). Although deaths attributed to illicit fentanyl had begun emerging by 2013, synthetic-opioid-related overdose mortality increased substantially from 2014 onward. Initially concentrated in the Northeast and Midwest, fentanyl-related mortality later expanded into the western United States (9). A fourth wave subsequently emerged involving increasing mortality associated with cocaine and psychostimulants, particularly methamphetamine, in combination with opioids. Between 2012 and 2018, cocaine-related mortality increased approximately threefold, from 1.4 to 4.5 deaths per 100,000 population, while psychostimulant-related mortality increased approximately fivefold, from 0.8 to 3.9 deaths per 100,000 population (10). This fourth wave is closely intertwined with the continuing fentanyl crisis because synthetic opioids are frequently involved in deaths attributed to cocaine and psychostimulants (10).

**Table 1:** Chronological overview of the four waves of the U. S. opioid overdose epidemic, summarizing the approximate time frame, predominant substances, and defining epidemiological characteristics associated with each stage of the evolving overdose crisis.

Phase	Time period	Key characteristics
Wave 1: Prescription Opioids	1990s–2011	Increased opioid prescribing and availability of high-dose and extended-release formulations. (2, 9)
Wave 2: Heroin	After 2010; peaked in 2017	Increased heroin use and mortality, including transitions from prescription opioids (9).
Wave 3: Synthetic opioids	2014–2021	Significant increase in synthetic-opioid mortality, initially concentrated in the Northeast and Midwest and later expanding westward (9).
Wave 4: Stimulant and polysubstance use	Emerging from approximately 2012 onwards; major increases documented around 2012–2018	Threefold increase in cocaine mortality and fivefold increase in psychostimulant mortality; increasing co-use of stimulants with heroin and synthetic opioids (10).

**Figure 2:**
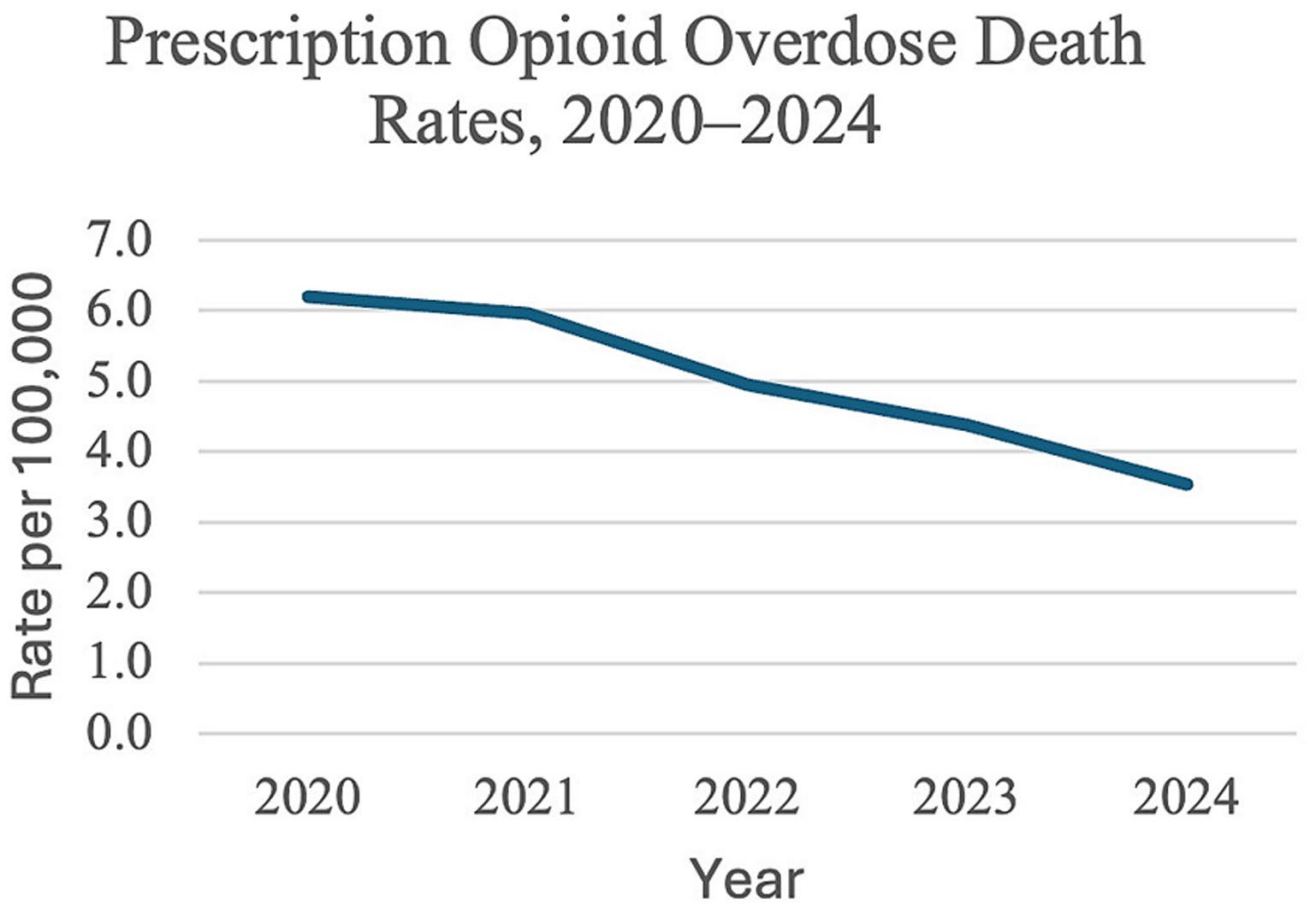
Trends in age-standardized prescription opioid overdose death rates (per 100,000 population) in the United States from 2020 to 2024. Rates were averaged across jurisdictions, with suppressed values excluded. Data obtained from the Centers for Disease Control and Prevention SUDORS dataset.

Florida was at the epicenter of the first wave, with its unregulated pain clinics distributing millions of prescription opioids (5). Rigg et al. (11) detailed how these clinics fueled the epidemic by granting easy access to high-dose opioids, often without adequate medical oversight. Even as these facilities were shut down, the demand for opioids shifted to illicit sources, further impeding efforts to control the crisis. Prescription opioid overdose death rates declined steadily from 2020 to 2024 (12) (Figure 2), denoting a reduction in mortality associated with prescription opioids during this period. Following these crises, the emerging concern in the modern era is the increase in prevalence of polysubstance use, particularly the method of using opioids in combination with other substances, which is commonly referred to as the fourth wave of the opioid epidemic (13).

### 3.2 Polysubstance use and emerging drug combinations

Polysubstance use has become a defining part of the OUD crisis in the United States, as many individuals with OUD do not use opioids by themselves but instead co-use them with other substances (14). Nearly half of drug overdose deaths in the United States now involve the use of more than one drug (15), showing how polysubstance use has become increasingly common and dangerous (Table 2). Rather than taking opioids alone, most people with OUD mix them with other central nervous system (CNS) depressants such as benzodiazepines, alcohol, and xylazine, as well as common stimulants like cocaine and methamphetamine. Nitazenes have also been encountered in polydrug mixtures within the illicit drug supply and are associated with a high risk of overdose and death (16). Nitazenes have been found mixed with substances such as fentanyl, heroin, and cocaine (17), and when used together, these drugs amplify one another’s effects, greatly raising the likelihood of overdose and dependence. The prevalence of polysubstance use continues to rise because people combine opioids with other drugs to increase the feeling of highness, reduce unwanted side effects, cope with mental or physical health issues, or unknowingly consume drugs that have been contaminated or altered in the illicit market. Although some individuals use opioids on their own, mixing them with other substances creates effects that are far more unpredictable and dangerous (18, 19).

**Table 2:** Key substances in polysubstance use.

Substance	Role	Risk
Fentanyl	Primary opioid	High overdose risk
Cocaine	Stimulant	Masks overdose
Benzodiazepines	Depressant	Respiratory suppression
Xylazine	Adulterant	Reduces naloxone effectiveness
Nitazenes	Synthetic opioids	Extreme potency

The table outlines the role of using different substances in polysubstance use with their associated risks. Fentanyl is the primary opioid with a high overdose risk, Cocaine is a stimulant that masks overdoses, and Benzodiazepines act as a depressant, which suppresses respiration. Xylazine is an adulterant known for reducing the effectiveness of naloxone, while Nitazenes are highly potent synthetic opioids.

A frequent example is the co-use of opioids and benzodiazepines, which deepens sedation and slows breathing (18–20). Benzodiazepines, often referred to on the street as “benzos” or “downers,” are commonly co-used by people with OUD because combining them with opioids can increase the sense of euphoria, and alcohol produces similar depressant effects when mixed with opioids (18, 21), even though their role is often under-recognized in routine surveillance data. Between 1999 and 2017, there were 399,230 poisoning deaths involving opioids, with 263,601 (66.0%) occurring among males and 204,560 (51.2%) among individuals aged 35 to 54 years (22). Alcohol involvement in all opioid overdose deaths rose in a nonlinear pattern from 12.4% in 1999 to 14.7% in 2017 (22). When broken down by opioid subtype, heroin and synthetic opioids showed the highest alcohol co-involvement in 2017, at 15.5 and 14.9%, respectively (22). Benzodiazepine involvement in all opioid overdose deaths also increased nonlinearly, from 8.7% in 1999 to 21.0% in 2017 (22). In 2017, benzodiazepines were involved in 33.1% of prescription opioid overdose deaths and 17.1% of synthetic opioid overdose deaths (22).

In recent years, xylazine, an animal sedative that is not approved for human use, has played an important role as a notably concerning adulterant found in fentanyl supplies (23). In an April 26, 2024, statement, DEA Administrator Anne Milgram noted that xylazine’s growing prevalence in the fentanyl supply is making fentanyl the deadliest drug threat facing the country even deadlier (24). The DEA has identified mixtures of xylazine and fentanyl in 48 out of 50 states. According to the DEA Laboratory System, in 2022, about 23% of fentanyl powder and 7% of fentanyl pills seized by the agency contained xylazine (25). In a statement, DEA Administrator Anne Milgram reported that in 2023, 30% of the fentanyl powder seized by the DEA contained xylazine, an increase from 25% in 2022 (24). The proportion of fentanyl pills testing positive for xylazine decreased slightly from 7% in 2022 to 6% in 2023 (24). Overall, the DEA seized 12,000 pounds of fentanyl powder and 79.5 million fentanyl pills in 2023 (24). Because xylazine is not an opioid, its presence in drug mixtures can lessen how well naloxone works, worsen tissue damage, and lead to more complicated withdrawal (26), which together heighten the overall health impact on individuals with OUD.

This pattern of depressant co-use highlights how the epidemiology of OUD is increasingly determined by complex combinations of substances rather than the use of a single drug. Some individuals also engage in “speedballing,” where an opioid or other depressant is combined with a stimulant like cocaine, producing opposing effects that can temporarily hide early signs of overdose (27). A growing concern in Nevada and other states is that many overdoses now involve a mix of substances, particularly opioids like fentanyl combined with stimulants such as methamphetamine. According to a 2022 state report, nearly one-third of overdoses in Nevada result from the use of both substances together (28). Some intentionally combine the two using a stimulant to stay alert or offset the heavy sedation from opioids, while others unknowingly take fentanyl mixed into what they assume is a stimulant-only dose (29). Also, the use of other opioids, including the nonmedical use of prescription painkillers and the synthetic opioid fentanyl, doubled between 2010 and 2020 (30).

**Figure 3:**
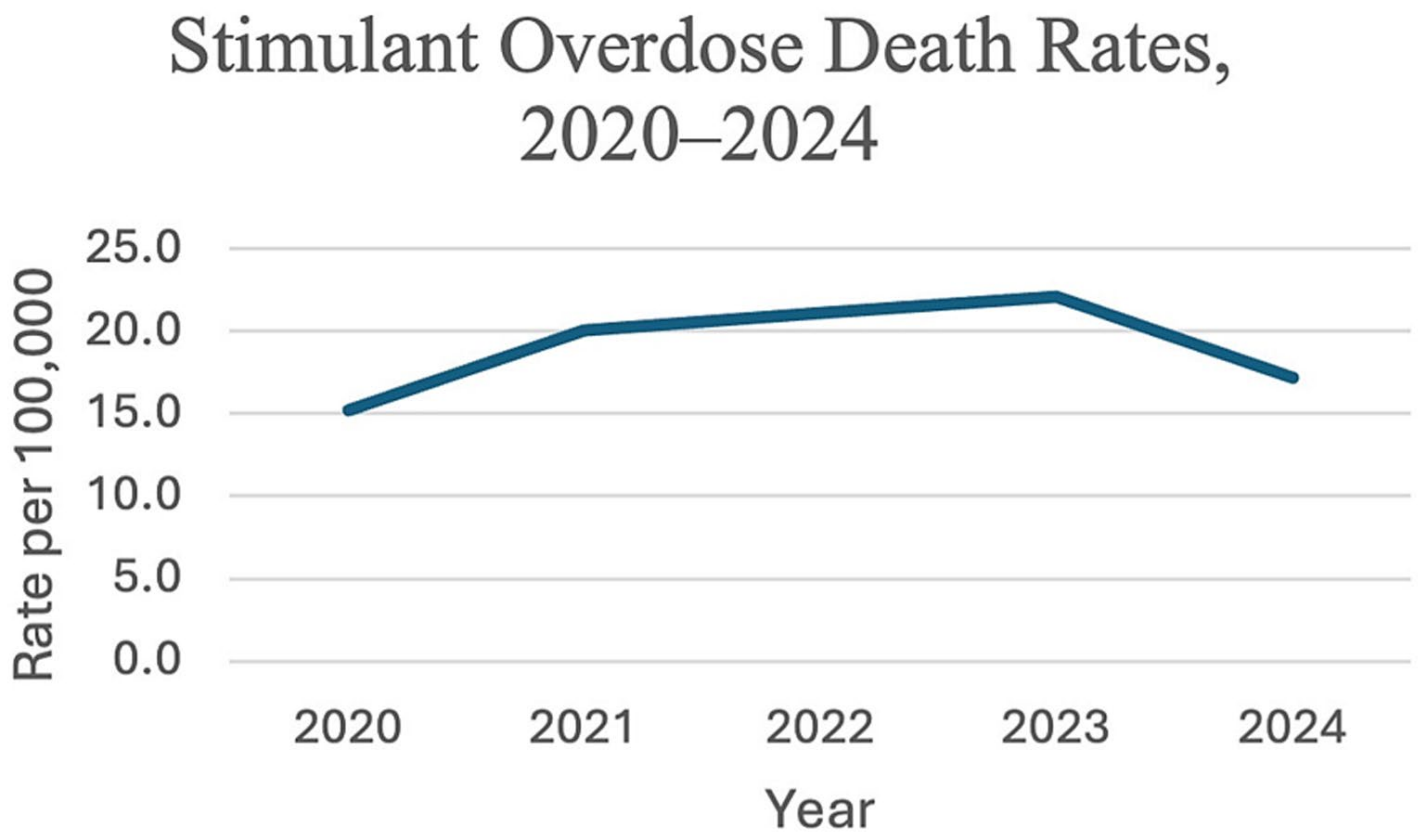
Trends in age-adjusted rate of drug overdose deaths involving any stimulants (per 100,000 persons) in the United States from 2020 to 2024. Rates were averaged across jurisdictions. Data obtained from the Centers for Disease Control and Prevention SUDORS dataset.

In several Western states, the co-use of opioids and stimulants has been a longstanding issue, with methamphetamine being more commonly involved than cocaine. The region has a known history of “goofball” use, a term like “speedball” referring to an injected mixture of heroin and methamphetamine (31). Reports of this practice have appeared in various Western cities since the early 2000s, showing that opioid-methamphetamine mixing has been an established pattern in the region for many years (32). For instance, a 2019 study of syringe exchange program (SSP) clients in Seattle found that 55% of participants reported using a goofball within the past 3 months, and the proportion identifying it as their main drug rose from 10% in 2017 to 20% in 2019 (31, 32). Similarly, a 2017 study using National HIV Behavioral Surveillance (NHBS) data from Denver reported that 28% of participants used goofballs, 43.9% used heroin and methamphetamine separately, and 24% used both (32, 33). Factors associated with the co-use of methamphetamine and opioids included younger age, homelessness, and recent incarceration (31–35). Stimulant overdose death rates increased from 2020 to 2023, followed by a slight decline in 2024 (12) (Figure 3), indicating a growing contribution of stimulants to overdose mortality.

### 3.3 Regional dynamics (South Florida case)

One of the major public health issues in Florida is opioids, accounting for the majority of overdose deaths in the state (36). In 2022, the state recorded 7,827 overdose deaths, ranking 18th nationally (36). Fentanyl was the major opioid contributor for these deaths (36). This opioid crisis imposed a substantial economic burden on the state, with illicit drug use resulting in approximately $6.4 billion in health care expenditure over 3 years. About $2.16 billion was paid in patient care by Medicare, and $1.36 billion was the expected amount paid by Medicaid and commercial insurance (36). South Florida is a major contributor in terms of opioid deaths. Counties like Broward, Palm Beach, and Miami-Dade account for a large share of deaths because of their vast populations (36). In South Florida, overdose deaths associated with prescription opioids reached their highest levels in the late 2000s, with oxycodone involved in most cases (37). More recent trends show a sharp rise in fentanyl-related deaths, highlighting the ongoing difficulty of controlling synthetic opioids (38). South Florida’s role in the opioid crisis provides important insights. During the peak of the “pill mill” era, unregulated pain clinics in the region distributed a disproportionately large share of the nation’s oxycodone supply (39).

According to Florida’s Medical Examiners Commission, 2,499 out of 2,882 cocaine-related deaths across the state involved cocaine used alongside other drugs (40). In Miami-Dade County, where there were about 36 cocaine-related deaths per month in 2016, the Medical Examiner’s Office reported that 84 individuals had both cocaine and carfentanil in their system at the time of death (40). During 2016–2017, carfentanil was responsible for a deadly outbreak in the U. S. with 1,190 deaths in Florida alone. Recent data suggests that there’s a resurgence of carfentanil-related deaths in Florida, where South Florida was the major contributor, contributing approximately 67.5% in 2016, 67.0% in 2017, and 68.8% during 2018–2023 (41). This recent stage of the overdose crisis, marked by a sharp rise in polysubstance use involving opioids, is commonly described as a “fourth wave.” Unlike earlier phases, it is not driven by a single drug but by the increasing prevalence of complex mixtures within the illicit drug supply (10).

Many people in Florida with OUD use stimulants to adjust the intensity of their high or simply because cocaine and methamphetamine are widely available in the region. According to reports from the Florida health authorities, the drugs responsible for the highest number of deaths were fentanyl (5,622), cocaine (2,598), methamphetamine (2,193), ethyl alcohol (1,364), benzodiazepines (900, including 528 alprazolam deaths), amphetamine (850), and fentanyl analogs (844). Fentanyl (90%), fentanyl analogs (84%), methamphetamine (75%), heroin (73%), cathinones (67%), cocaine (66%), methadone (59%), xylazine (57%), and mitragynine (56%) were identified as causing death in more than 50 percent of the cases in which these substances were present (42). These patterns show that stimulants are causing thousands of deaths in Florida, most often alongside opioids as part of polysubstance use. State data consistently indicate that when stimulants or opioids are detected in a fatal case, they usually play a major role in the outcome.

Fentanyl and its analogs now account for a majority of opioid-related overdose deaths in the region (38). Legislative measures such as the implementation of the Prescription Drug Monitoring Program (PDMP) and stricter regulations on pain clinics significantly reduced prescription opioid misuse (43). Florida’s 3-day opioid prescription law (HB21) made a significant impact on the reduction in both the number of opioid units dispensed and total morphine milligram equivalents (MMEs) prescribed for acute pain patients (44). Expanding community outreach programs, enhancing access to treatment, continuous monitoring, faster public health response, and addressing socioeconomic disparities are critical for mitigating the ongoing crisis. Future policies require responses addressing not only opioids like fentanyl but also the stimulants that frequently accompany them.

### 3.4 Demographic and structural risk factors

The OUD disproportionately affects specific demographic groups by age, gender, race, and socioeconomic status (Table 3). In 2015, Anne Case and Angus Deaton proposed the “deaths of despair” theory, ascribing the rise in opioid-related deaths to factors such as widespread poverty, income inequality, job loss from declining labor markets, limited social support, restricted access to healthcare, and increasing social isolation (45). The 2024 data show clear demographic differences in opioid-related deaths across the United States, with a particularly high burden among Hispanic populations. Nationwide, there were 7,899 deaths among Hispanic individuals, compared to 518 among Asians, 845 among American Indian or Alaska Native populations, 70 among Native Hawaiian/Pacific Islanders, and 824 among individuals of multiple races, highlighting unequal impacts across groups. At the state level, Florida reflects a similar national trend, reporting 487 deaths among Hispanic individuals, along with 10 Asian deaths and 10 American Indian or Alaska Native deaths, while data for Native Hawaiian/Pacific Islander and multiple race groups were not reported. These findings indicate that although Florida contributes significantly to the national Hispanic burden, missing data for some populations may limit a full understanding of demographic disparities within the state (46).

**Table 3:** Major risk factors for OUD.

Category	Key characteristics
Biological variation	Genetic predisposition, dopamine pathway (57)
Psychological	Mental Health disorders, trauma (47)
Social	Poverty, unemployment, isolation (45, 56)
Medical	Opioid prescribing, chronic pain (5)

The table summarizes the key biological, psychological, social, and medical risk factors for OUD. Biological variations include genetic predisposition and dopamine pathways; mental health disorders and trauma are major psychological contributors. Social characteristics such as poverty, isolation, and unemployment can further increase vulnerability. While medical factors, including opioid prescribing and chronic pain conditions, play a significant role in the initiation and progression of OUD.

Age is one of the important factors to be considered when studying OUD trends, as the generational impact of the disease can be revealed. Young adults aged 18–35 exhibit the highest prevalence rates. The COVID-19 pandemic has contributed to the soaring numbers of opioid-related deaths, and teen death counts were found to be higher than those of adults (47). In an environment, like schools and colleges, where opioid usage may be normalized, peer pressure and stress can be a major reason for the increasing rates of opioid use among young adults. However, teenage opioid use is a significantly smaller value compared to opioid misuse among the middle-aged and older populations (48). As age increases, the likelihood of developing chronic diseases increases. Patients diagnosed with chronic diseases are likely to be prescribed opioids to effectively manage the pain. About 35% of older adults who were prescribed opioids for pain management have reported misusing their prescriptions (49).

Gender differences are notable; men are more likely to misuse opioids through illicit use and alcohol or stimulant co-use, whereas women tend to progress more rapidly from prescription use to dependence and face additional challenges, including pregnancy-related complications and neonatal abstinence syndrome (48). Over the past decades, the rate of OUD has risen by 642% among women, compared to a 439% increase among men (50). This data can be ascribed to a combination of biological, medical, and community factors. For example, women are more likely to be prescribed opioids for chronic pain conditions and tend to transition from prescription use to dependence more quickly than men. Opioids are commonly used in managing chronic conditions such as Alzheimer’s disease or osteoporosis, and women are often prescribed higher doses compared to men. In addition to chronic pain, women are more susceptible to mental health disorders and are therefore more likely to use prescription opioids (51). Higher opioid doses and prolonged opioid use for pain management or relief of mental health symptoms may pose a greater risk for women, as discontinuation can be difficult and may increase the likelihood of misuse or overdose (52).

In addition to sex and socioeconomic factors, ethnicity also influences the epidemiology of opioid use disorder (OUD). Although OUD was initially most prevalent among Caucasians, particularly young White men, its prevalence has increased substantially among African American populations (53). Racial disparities in medical care contribute to differences in OUD treatment and outcomes (54). Differences in access to support for OUD and the prescribing of opioids for certain chronic conditions may contribute to additional problems, ultimately reducing the quality of life for many individuals (55). The lack of fair treatment in the healthcare system can increase the risk of new illicit synthetic drugs emerging, which is a pattern that can be connected to populations of lower socioeconomic status, as well.

Social and economic factors, including poverty, unemployment, and restricted educational attainment, significantly increase the risk of developing OUD (56). These disparities are especially evident in rural areas and economically disadvantaged communities. The study by Rigg et al. (11) further highlighted how economic strain and limited access to medical care contributed to increased opioid misuse, particularly through unregulated pain clinics. Although the risk of chronic disease and inadequate treatment is common, illicit opioid use is especially prominent within this population (11).

Prescription practices have played a major role in the development of OUD. From the late 1990s through the early 2010s, aggressive promotion of opioids for pain management normalized their use and contributed to widespread misuse. During this period, pain clinics, often called “pill mills,” became increasingly common, especially in South Florida, further worsening the epidemic (5). The development of OUD is also shaped by complex interactions between genetic predispositions and environmental stressors. Research indicates that OUD has a moderate to high heritability component, with twin and family studies identifying specific genetic factors linked to increased risk (57). Environmental elements such as childhood trauma, unstable housing, and social determinants of health also have important roles in heightening susceptibility to OUD (4).

Genetic susceptibility is a key factor in addiction vulnerability. A major focus of neuropsychiatric research is to determine why some individuals are more prone to addiction than others. As with many psychiatric conditions, the risk of OUD arises from complex interactions between genetic predispositions and environmental influences. Drugs of abuse, including opioids, act on the brain’s reward system, which is largely driven by the neurotransmitter dopamine (58). The functioning of this system is shaped by both inherited genetic factors and environmental stressors. Emerging evidence has identified new genetic mechanisms that contribute to addiction risk. Recent research has shown that the integration of a human endogenous retrovirus element (HERV-K HML-2) within genes involved in dopaminergic signaling occurs more frequently in individuals with substance use disorders. This integration has been linked to disruptions in dopamine regulation and a higher vulnerability to drug addiction, highlighting the complex molecular pathways through which genetic factors may influence opioid dependence (59, 60).

### 3.5 Treatment access and gaps

Effective management of OUD requires a combination of prevention, treatment, and harm reduction strategies (Table 4). Evidence-based treatments such as methadone and buprenorphine have been shown to reduce cravings and withdrawal symptoms, supporting long-term recovery (61). Naltrexone, another medication for OUD, blocks the euphoric effects of opioids, helping prevent relapse (62). A study published in the JAMA Health Forum examined the trends in methadone and buprenorphine use for OUD among Medicaid patients from 1999 to 2020 and found that both medications have increased over time. Although a large portion of people with OUD did not receive medication-based treatment, which shows an ongoing gap in care (63).

**Table 4:** Selected strategies addressing opioid use disorder, treatment access, and associated harms.

Strategy	Examples	Purpose
Medication-assisted treatment (MAT)	Methadone and buprenorphine	Support treatment retention, reduce opioid use and cravings, and lower the risk of opioid-related morbidity and mortality (61)
Harm Reduction	Naloxone, syringe Programs	Reverse opioid overdose, reduce overdose mortality and injection-related harms, and connect individuals with healthcare and treatment services (62, 64)
Policy and monitoring	Prescription drug monitoring programs (PDMPs)	Support safer prescribing practices and (65) identify potentially inappropriate or high-risk prescribing patterns
Technology-based intervention	Artificial intelligence–based risk detection	Identify individuals at elevated risk and support timely clinical assessment and early intervention (74)

The table summarizes the key strategies used to address OUD. Treatment approaches like methadone and buprenorphine help in reducing mortality of OUD. Harm reductions include wide availability of naloxone and syringe programs, and key policy implementations like PDMPs aid in prevention of overdoses and reduction of overprescribing. Emerging technologies like artificial intelligence help in risk detection by providing early intervention of OUD.

Harm reduction approaches, including naloxone distribution and syringe exchange programs, have proven critical in preventing overdose deaths and reducing disease transmission. Public health initiatives like prescription drug monitoring programs (PDMPs) have also been effective in reducing overprescription (64). Preventing OUD and helping people recover from it means taking a wide-ranging approach, one that focuses on stopping misuse early and supports individuals throughout the long process of treatment and recovery. Many effective OUD treatments require long-term engagement (often well beyond 30 days), yet a large share of people who need help never enter treatment (65).

In 2023, according to the Substance Abuse and Mental Health Services Administration (SAMHSA), only about one in four people in the United States who needed treatment for substance use were able to receive it (66). Many people face barriers such as high costs, not having enough time, and fear they might lose their jobs if they seek longer-term treatment. According to Fu et al. (67), 129 patients were assessed for medication-assisted treatment (MAT). About 53% received MAT, 35% were given only a referral, and 12% declined any form of intervention. The median age of participants was 36 years (interquartile range [IQR], 28–46 years). The group was largely male (73%), single (65%), and white (73%), with most individuals being unemployed (57%), insured through public programs (55%), and without a primary care physician (58%). Clinically, the majority presented opioid withdrawal (62%), followed by intoxication (15%), while 23% had other presenting concerns. In addition, 51% of patients were discharged with a naloxone kit. Among those who received treatment, most were started on buprenorphine at doses of 4 mg or less (54%), and only 6% required additional dosing (67).

Another major hurdle in addressing OUD is the cost of treatment. Many people simply cannot afford the services they need, even though Medicaid pays for a large portion of substance use treatment in the United States. Out-of-pocket expenses still make treatment unaffordable for many individuals. According to Dowell et al. (68), the use of treatment among adults with OUD differed notably across demographic and clinical groups. Adults aged ≥50 years had a lower treatment rate (44.9%) compared with younger groups (58.9–67.8%). Treatment was more common among non-Hispanic white adults (60.3%) than among non-Hispanic black (43.8%) and Hispanic (45.7%) adults. Individuals with severe OUD were more likely to receive treatment (53.0%) than those with mild or moderate OUD (20.5%). Additionally, higher treatment rates were observed among adults who were employed (63.1%), had a history of arrest and booking (66.3%), or reported using illicit drugs other than opioids (61.2%), highlighting disparities shaped by socioeconomic and behavioral factors (68).

Improving access to affordable care, whether through better insurance coverage or subsidized programs, can make a meaningful difference. Flexible treatment options can also help. For example, outpatient and telehealth-based OUD programs allow people to keep working while getting support. Newer policies that provide job-protected medical leave for addiction treatment directly reduce the fear of losing employment while in care. Guaranteeing paid, job-protected leave could remove major barriers and motivate many more people to seek treatment. Evidence from states that have implemented paid medical leave, including leave for substance use treatment, shows that more individuals enter care when they no longer must choose between their health and their income (69). Overall, making OUD treatment affordable, accessible, and flexible enough to fit around work responsibilities is crucial for helping more people receive the support they need.

Making naloxone widely available now, even sold over the counter and teaching people how to use it, has already saved many lives, and continued investment in these harm-reduction strategies remains crucial (70). Building up community support services, such as recovery coaching, mental health care, and job training for people in recovery, can also help individuals stay on track and maintain long-term stability (71). Beyond expanding treatment options, education and technology also play important roles in addressing the opioid crisis like public awareness efforts can help prevent misuse in the first place and reduce the stigma that prevents individuals from seeking assistance. For example, the “Start Talking” campaign in the United States promotes open discussions between parents and children about the risks of opioids. Similarly, government agencies such as the Centers for Disease Control and Prevention (CDC) and the SAMHSA have implemented nationwide initiatives to educate the public on opioid safety and the proper disposal of unused medications (72). Such efforts not only highlight how addictive potential of opioids, but also remind people that OUD is a medical condition with real treatment options, a message that helps push back against the stigma that often stops individuals from reaching out for support. Law enforcement has also played a role in spreading awareness. The DEA’s “One Pill Can Kill” campaign, for example, focuses on educating communities about the dangers of counterfeit pills laced with fentanyl and emphasizes the importance of using medications only when they come from licensed and trusted medical sources (73). These kinds of efforts are important because they help shift public attitudes and behaviors surrounding opioid use.

### 3.6 Emerging interventions (AI, harm reduction)

Meanwhile, development in newer technologies and AI are creating new opportunities like early detection, diagnosis and treating OUD. Healthcare teams are beginning to use machine learning to examine patient patterns and identify signs of OUD risk much earlier, and there is a growing need for these tools to be used efficiently and expanded as technology advances (74). As improved technologies continue to emerge, integrating them into routine care can strengthen early detection, support better decision-making, and enhance the overall prevention and treatment of OUD (74).

One recent study reported that a machine learning (ML) application called MedAware was evaluated for its ability to identify patients at higher risk of OUD by analyzing patient records. The findings indicated that ML can provide clinicians with reliable alerts regarding a patient’s level of risk (75). These AI-based tools could be used in clinics and pharmacies to flag patients who may be misusing opioids or at risk of an overdose, allowing providers to offer support sooner and make safer prescribing decisions. A study led by Dr. Majid Afshar, developed an AI-based screening tool that analyzed electronic health records (EHR’s) to identify patients at risk for OUD, subsequently recommending specialist care (74). This clinical trial involved more than 51,000 patients, and the AI system demonstrated performance comparable to traditional providers’ assessment in identifying candidates for OUD (74). The most remarkable thing is that the patients who were screened by AI were 47% less likely to be readmitted within 30 days, which saved an estimated $6,800/patient in health care costs (76, 77).

Although AI-based screening may support earlier identification of patients at risk for OUD, the current evidence remains limited. Before widespread clinical implementation, these tools require external validation across diverse populations, assessment of algorithmic bias and false-positive rates, protection of patient privacy, and evaluation of their effects on clinical workflows and patient outcomes. Along with expanding treatment access, improving awareness, and incorporating AI without risks, several additional steps can strengthen our overall response to OUD. Combining affordable, judgment-free treatment with strong prevention efforts and modern technology offers the best path toward reducing the impact of OUD. Successes already seen in the real world, from workplaces adopting recovery-supportive policies to AI tools helping identify people at risk, show that these approaches can make a difference (74). By expanding these efforts and continuing to innovate, we can lower barriers to care, identify problems earlier, and prevent overdose deaths, helping to turn the tide of the opioid crisis.

## 4 Discussion

This review indicates that the contemporary opioid crisis is better understood as a dynamic polysubstance crisis than as a sequence of separate drug-specific waves. Illicit fentanyl, stimulants, benzodiazepines, xylazine, nitazenes, and carfentanil increasingly overlap through intentional co-use and unintentional contamination. The four-wave model remains historically useful, but it does not adequately guide responses to substances that coexist and change rapidly. We therefore propose an adaptive polysubstance response framework organized around four steps: detecting changes in the drug supply, identifying the affected populations and exposure pathways, activating predefined interventions, and evaluating whether those interventions reduce harm.

Drug-supply instability requires surveillance capable of detecting threats before they become established in mortality data. Local health departments should integrate emergency-department, emergency medical services, medical-examiner, toxicology, and community drug-checking data. Laboratories should use standardized toxicology panels, retain specimens for confirmatory testing when unfamiliar substances are suspected, and clearly distinguish substances that were detected from those determined to have contributed to death. Detection of an unusual overdose cluster or emerging substance should trigger rapid alerts to hospitals, harm-reduction programs, treatment providers, and affected communities. Performance should be measured using the time from detection to alert and from alert to intervention, rather than the number of reports produced. This approach is consistent with the CDC’s (centers for disease control) and prevention emphasis on linking biosurveillance, nonfatal-overdose, toxicology, and mortality data (78).

The distinction between intentional co-use and unintentional exposure should also determine the intervention. Toxicological co-detection alone cannot establish intent; consequently, clinicians and researchers should combine toxicology results with confidential, non-punitive interviews about the substances individuals intended to consume. Patients intentionally combining opioids and stimulants should be offered medication for OUD together with integrated treatment for stimulant use and co-occurring mental-health conditions. Contingency management can be incorporated when clinically appropriate for stimulant use disorder (79–82). Unintentional exposure requires a different response: accessible drug checking, fentanyl test strips where applicable, targeted alerts describing the affected product and location, and naloxone provision. Treatment programs should measure both opioid-treatment retention and changes in stimulant use rather than treating opioid abstinence as the only meaningful outcome.

The persistent underuse of methadone and buprenorphine requires redesign of treatment delivery, not simply encouragement to seek care. Emergency departments, hospitals, primary-care practices, syringe-service programs, and mobile clinics should offer same-day treatment initiation or direct warm handoffs to a provider who can continue medication. Before discharge, patients should receive naloxone, a bridge medication when clinically appropriate, a confirmed follow-up appointment, and assistance with transportation or insurance enrollment. Clinics should provide walk-in access, evening or weekend hours, flexible follow-up, and telehealth when clinically and legally appropriate. Insurers and Medicaid programs should reimburse treatment initiation and patient navigation while removing unnecessary prior-authorization and cost-sharing barriers. Medication should not be withheld solely because a patient continues to use other substances or declines counseling. These measures reflect current low-barrier and linkage-to-care recommendations (83, 84).

Treatment continuity is especially important during incarceration and re-entry. Correctional facilities should continue medication for people already receiving it, allow treatment initiation during incarceration, and arrange community appointment before release. Individuals should leave with naloxone and a medication-continuity plan rather than a list of providers to contact independently. Treatment performance should be reported as a cascade, OUD identification, medication initiation, follow-up, and retention and stratified by race, age, insurance status, and geography. This would identify where patients are lost from care and permit resources to be directed toward documented gaps.

The involvement of fentanyl and xylazine also requires modification of overdose and harm-reduction services. Naloxone kits should be placed directly in emergency departments, pharmacies, treatment programs, correctional-release programs, shelters, and syringe-service programs. Distribution should include practical instruction in overdose recognition, naloxone administration, rescue breathing, and activation of emergency services. Because naloxone does not reverse xylazine’s non-opioid effects, suspected overdoses require naloxone for possible opioid involvement together with respiratory support and emergency care (85). Harm-reduction programs should also provide low-barrier wound assessment, basic wound supplies, infection referral, HIV and hepatitis testing, drug checking, and direct access to OUD treatment. Mobile services should be used where fixed-site programs are inaccessible.

Prescription drug monitoring programs should be retained as clinical safety tools but should not remain the central response to a fentanyl-dominated illicit market. A high-risk prescribing pattern should trigger a patient-centered assessment of pain, overdose risk, and possible OUD, not abrupt medication discontinuation or dismissal from care. When OUD is identified, the response should include treatment initiation and naloxone provision. Program success should be evaluated using overdose, treatment linkage, and patient-safety outcomes rather than reductions in prescribing alone. CDC guidance similarly cautions against applying prescribing recommendations as inflexible limits (86).

South Florida provides a practical setting in which to apply this framework. Miami-Dade, Broward, and Palm Beach counties should establish a shared surveillance and response protocol linking hospitals, emergency services, medical examiners, laboratories, treatment providers, and harm-reduction organizations. When surveillance identifies an emerging substance or geographic cluster, predefined actions should include targeted naloxone deployment, mobile treatment and wound-care services, drug checking, and clinician alerts. Resources should be allocated using population-adjusted overdose rates and treatment-access gaps rather than absolute death counts. Regional performance could be measured through detection-to-alert time, naloxone coverage, same-day treatment initiation, 30- and 90-day medication retention, nonfatal overdose recurrence, and mortality.

AI-assisted screening may support this system, but it should not be implemented solely because a model predicts risk accurately. Before deployment, models should be externally and prospectively validated, compared across demographic groups, and evaluated for false-positive and false-negative results. Every alert should receive human clinical review and lead to an offer of care rather than punitive action. Health systems should use minimum necessary data, access controls, audit logs, and monitoring for model drift or unauthorized use. The relevant endpoint is not the number of patients flagged but whether AI increases voluntary treatment initiation and improves outcomes without worsening disparities.

## 5 Conclusion

The opioid crisis has evolved into a dynamic polysubstance crisis shaped by an unstable illicit drug supply, structural vulnerability, and uneven institutional capacity. Reducing overdose mortality requires equitable access to methadone and buprenorphine, expanded naloxone distribution, community-based harm reduction, and timely toxicology surveillance. Prescription-monitoring policies and AI-assisted screening may support this response but should not replace established interventions. Ultimately, progress will depend on how rapidly healthcare and public-health systems can identify emerging threats and deliver accessible, evidence-based care.

### 5.1 Limitations

This review has several limitations. Although a structured, PRISMA-informed search was conducted, the findings were synthesized narratively because of substantial heterogeneity in study designs, populations, geographic settings, data sources, and outcome definitions. Accordingly, pooled estimates of OUD prevalence, overdose mortality, treatment access, and intervention effectiveness were not calculated. Searches involving Google Scholar and government websites may also be difficult to reproduce fully, and no formal risk-of-bias assessment was performed.

Most included evidence was observational or derived from secondary data and therefore cannot establish causality. Incomplete documentation, inconsistent toxicology testing, and variation in death-certificate coding may have contributed to underreporting or misclassification. Toxicological co-detection also cannot determine whether polysubstance exposure was intentional or establish the independent contribution of each substance to death. Finally, the primary focus on the United States and South Florida limits generalizability, while surveillance and publication delays may not capture the rapidly changing illicit drug supply. The four-wave framework should therefore be interpreted as a historical organizing model rather than as a set of fixed or mutually exclusive stages.
